# *Staphylococcus epidermidis* Biofilm-Released Cells Induce a Prompt and More Marked *In vivo* Inflammatory-Type Response than Planktonic or Biofilm Cells

**DOI:** 10.3389/fmicb.2016.01530

**Published:** 2016-09-27

**Authors:** Angela França, Begoña Pérez-Cabezas, Alexandra Correia, Gerald B. Pier, Nuno Cerca, Manuel Vilanova

**Affiliations:** ^1^Laboratory of Research in Biofilms Rosário Oliveira, Centre of Biological Engineering, University of MinhoBraga, Portugal; ^2^Departamento de Imuno-Fisiologia e Farmacologia, Instituto de Ciências Biomédicas de Abel Salazar, Universidade do PortoPorto, Portugal; ^3^Instituto de Investigação e Inovação em Saúde, Universidade do PortoPorto, Portugal; ^4^Instituto de Biologia Molecular e Celular, Universidade de PortoPorto, Portugal; ^5^Division of Infectious Diseases, Department of Medicine, Brigham and Women's Hospital/Harvard Medical SchoolBoston, MA, USA

**Keywords:** *S*. *epidermidis*, biofilms, biofilm-released cells, splenocytes transcriptome, pro-inflammatory cytokines, tissue colonization

## Abstract

*Staphylococcus epidermidis* biofilm formation on indwelling medical devices is frequently associated with the development of chronic infections. Nevertheless, it has been suggested that cells released from these biofilms may induce severe acute infections with bacteraemia as one of its major associated clinical manifestations. However, how biofilm-released cells interact with the host remains unclear. Here, using a murine model of hematogenously disseminated infection, we characterized the interaction of cells released from *S. epidermidis* biofilms with the immune system. Gene expression analysis of mouse splenocytes suggested that biofilm-released cells might be particularly effective at activating inflammatory and antigen presenting cells and inducing cellular apoptosis. Furthermore, biofilm-released cells induced a higher production of pro-inflammatory cytokines, in contrast to mice infected with planktonic cells, even though these had a similar bacterial load in livers and spleens. Overall, these results not only provide insights into the understanding of the role of biofilm-released cells in *S. epidermidis* biofilm-related infections and pathogenesis, but may also help explain the relapsing character of these infections.

## Introduction

*Staphylococcus epidermidis* is one of the most important etiological agents of device-associated infections due to its ability to adhere and form biofilms on the surface of indwelling medical devices (Vuong and Otto, 2002; Otto, 2009). When compared to planktonic cells, *S. epidermidis* cells within biofilms are known to be more tolerant to several classes of antibiotics (Cerca et al., 2005), as well as to the host immune effectors (Cerca et al., 2006; Kristian et al., 2008). Biofilms represent therefore a common cause of recurrent and relapsing infections (Costerton et al., 1999). Consequently, removal of the infected devices is often required to resolve these infections (von Eiff et al., 2002), which results in increased morbidity and, occasionally, mortality among infected patients (Otto, 2009). Due to the enormous impact of *S. epidermidis* biofilm-related infections on human health, the mechanisms underlying biofilm formation have been extensively studied in the last decades. It is currently accepted that biofilm formation involves three main stages: (1) initial adhesion, (2) maturation, and (3) disassembly (Otto, 2012). The later refers to the release of bacterial cells from the biofilm to the surrounding environment, and is the least understood stage of the biofilm lifecycle (Boles and Horswill, 2011). Importantly, biofilm disassembly has been associated with the emergence of severe acute infections such as bacteraemia (Wang et al., 2011) and the embolic events of endocarditis (Pitz et al., 2011). However, despite its clear importance in the clinical setting, little is known regarding the phenotype or interaction of these cells with the host immune system. In the first stages of biofilm formation, planktonic bacteria attached to medical devices undergo several physiological modifications that lead to the biofilm phenotype (Yao et al., 2005). Thus, it was thought that after disassembly biofilm-released cells would quickly revert to the planktonic phenotype (Kaplan, 2010; Chua et al., 2014). However, recent reports have shown that cells released from *Pseudomonas aeruginosa* (Rollet et al., 2009; Li et al., 2014), *Streptococcus mutans* (Liu et al., 2013), and *Streptococcus pneumoniae* (Marks et al., 2013) biofilms present features distinct from either the biofilm or planktonic phenotypes, showing higher virulence potential. Chua and collaborators showed that *P. aeruginosa* biofilm-released cells, when compared with their planktonic or biofilm counterparts, present higher expression level of genes associated with the bacterium virulence, namely Type 2 Secretion System (TSS) and T3SS *psc* gene and, more important, they showed that these genes are essential in eliciting full virulence against macrophages and in the rapid killing of *Caenorhabditis elegans* (Chua et al., 2014), respectively. In the case of *S. epidermidis*, it is only known that biofilm-released cells present higher tolerance than planktonic and biofilm cells to antibiotics (Franca et al., 2016). However, their full virulence potential remains unclear. A comprehensive analysis of the interaction between biofilm-released cells and the host would clarify their role in the pathogenesis of biofilm-related infections, and help to prevent the pathologic events associated with biofilm cells dissemination. Therefore, herein, a murine model of hematogenously disseminated infection was used to evaluate the capacity of *S. epidermidis* biofilm-released cells to (1) induce changes in the transcriptome of murine immune cells within the spleen, (2) stimulate the production of pro-inflammatory cytokines, and (3) colonize and persist in murine organs. Our results showed that *S. epidermidis* biofilm-released cells induce a prompt and more marked inflammatory-type response than do their planktonic or biofilm counterparts. In addition, these findings showed that particular properties of the biofilm-released cells need to be taken into account to efficiently target and treat acute infections originating from *S. epidermidis* biofilms.

## Materials and methods

### Ethics statement

This study was performed in strict accordance with the recommendations of the European Convention for the Protection of Vertebrate Animals used for Experimental and Other Scientific Purposes (ETS 123), the 86/609/EEC directive and Portuguese rules (DL 129/92). All experimental protocols were approved by the competent national authority (Direcção-Geral de Veterinária), document 023517 (2010.11.25).

### Mice

Female BALB/c mice, 8–12 weeks old, were purchased from Charles River (Barcelona, Spain) and kept under specific-pathogen-free conditions at the Animal Facility of the Instituto de Ciências Biomédicas Abel Salazar, Porto, Portugal. Mice were maintained in individually ventilated cages (5 animals per cage) with corncob bedding, and under controlled conditions of temperature (21 ± 1°C), relative humidity (between 45 and 65%) and light (12 h light/ dark cycle). Mice had *ad libitum* access to food and water. Hiding and nesting materials were provided for enrichment. All procedures such cage changing, water and food supply, as well as intravenous injections were always performed during the day cycle (between 7 and 19 h).

### Bacteria and growth conditions

The biofilm forming strain *S. epidermidis* 9142, isolated from a blood culture (Mack et al., 1994), was used in this study. A single colony, from a Tryptic Soy Agar (TSA) plate, was inoculated into 2 mL of Tryptic Soy Broth (TSB, Liofilchem, Teramo, Italy) and incubated overnight at 37°C with shaking at 120 rpm. A suspension with ~1 × 10^8^ CFU/mL, prepared by adjusting the optical density (at 640 nm) of the overnight culture to 0.25 ± 0.05, was used to start both planktonic and biofilm cultures. For planktonic cultures 150 μL of 1 × 10^8^ CFU/mL bacterial suspension was inoculated into 10 mL of TSB supplemented with 0.65% (v/v) glucose (TSB_0.65%G_) and incubated for 24 h at 37°C under agitation at 120 rpm. Biofilms were grown in 24-well plates made of polystyrene plastic (Orange Scientific, Braine-l' Alleud, Belgium) by inoculating 15 μL of the 1 × 10^8^ CFU/mL bacterial suspension into 1 mL of TSB_0.65%G_, then incubating at 37°C with agitation at 120 rpm. After 24 h of growth, biofilms were washed twice with apyrogenic Phosphate Buffered Saline (PBS, Gibco, MD, USA), 1 mL of fresh TSB_0.65%G_ was carefully added and biofilms allowed to grow, under the same temperature and agitation conditions, for additional 24 h. Biofilm-released cells, (i.e., the cells in the biofilm bulk-fluid), were collected as described before (Franca et al., 2016) from 12 originating biofilms and pooled together to decrease variability inherent to biofilm growth (Sousa et al., 2014). Four biofilms were washed twice with apyrogenic PBS, disrupted and also pooled together to reduce variability. Planktonic (4 mL of culture), biofilm and biofilm-released cells were then harvested by centrifugation, suspended in 4 mL of apyrogenic PBS (Gibco, MD, USA) and sonicated for 10 s at 18 W (Branson model W 185 D, Heat Systems Ultrasonics, CT, USA) in order to dissociate cell clusters. Cells viability was not reduced by this procedure as determined previously by CFU counting and propidium iodide incorporation (Cerca et al., 2011).

### Murine model of hematogenously disseminated infection

The inoculum of each of the bacterial populations was adjusted by flow cytometry to 5 × 10^8^ total cells/mL, using SYBR Green (LifeTechnologies, MD, USA)/propidium iodide (Sigma, MO, USA) staining, as optimized before (Cerca et al., 2011). The number of cultivable cells was assessed by CFU counting. Adult mice, randomly allocated to each experimental group, were injected intravenously in the lateral tail vein, with the support of a restrainer, with 0.2 mL of 5 × 10^8^ of planktonic, biofilm or biofilm-released cell suspensions. Control mice were injected intravenously with 0.2 mL of apyrogenic PBS. Sample size was determined based on the results of preliminary experiments. It was not possible to perform subsequent mouse studies in a blinded fashion. In order to address the alterations occurring during the acute phase of infection, the parameters evaluated in this study were assessed 2, 6, or 14 h after challenging the three *S. epidermidis* populations.

### Serum collection and bacterial load determination in organs

Two, 6, and 14 h post-infection, mice were anesthetized with isoflurane (Abbott laboratories, IL, USA) for terminal blood collection, and then euthanized by cervical dislocation. For serum collection, mouse blood was drawn through the retro-orbital route, incubated overnight at 4°C, and then centrifuged for 15 min at 4°C at 16,000 *g*. Serum was then transferred into a new tube and stored at −80°C until further use. Livers and spleens were aseptically removed and immediately transferred into tissue grinders with, respectively, 3 or 1 mL of PBS. Tissues were homogenized and quantitatively cultured on TSA plates. At all times during the procedure, samples were kept on ice. This experiment was performed 1 (for biofilms cells at all time points) to 3 (planktonic and biofilm-released cells, 6 h time point) independent times, with at least 5 animals per infected group.

### Cytokines and chemokines quantification

Two, 6, and 14 h post-infection, the levels of the cytokines IL-6, TNF-α and the chemokines CXCL1 (KC), CCL2 (MCP-1), CCL3 (MIP-1α), CCL4 (MIP-1β) in mouse serum were quantified in a Bio-Plex® 200 using the kit Magnetic Custom Multiplex Bio-Plex Pro Mouse Cytokine Group I assay (Bio-Rad, CA, USA). The procedure was performed following the manufacturer's instructions. This experiment was performed 1 (all populations at 6 and 14 h time point) to 2 (all populations at 2 h time point) independent times, with at least 5 animals per infected group.

### Microarray analysis of mouse splenocytes

Spleen cells plays a major role in host immune response to blood-born pathogens, working in concert to activate mechanisms required for successful resolution of infection. Hence, in order to address the mechanisms activated during the first contact with the different *S. epidermidis* populations, the transcriptome of splenocytes was analyzed, by microarrays, 2 h after challenge. In brief, spleens were aseptically removed, transferred to 60 mm diameter sterile Petri dishes with 9 mL apyrogenic PBS and immediately placed on ice. Thereafter, using two sterile frosted glass slides, spleens were completely homogenized. The suspension was then passed through a sterile column of glass wool to remove fibrous tissue, the number of cells counted by flow cytometry, and 5 × 10^6^ splenocytes harvested by 5 min centrifugation at 1200 rpm at 4°C. Cell pellets were immediately suspended in RLT buffer (QIAGEN, Heidelberg, Germany) and stored at −80°C until the next day. Total RNA was then isolated using the RNeasy Mini Kit (QIAGEN) following the manufacturer's instructions. Concentration and purity was determined using a NanoDrop1000 and integrity was confirmed using an Agilent 2100 Bioanalyzer (Agilent Technologies, CA, USA). RNA integrity number values were above 8.5 for all samples. This experiment was performed once with 2 (control and planktonic cells) to 3 (biofilm and biofilm-released cells) animals per group.

Transcription levels in mouse splenocytes were determined using Affymetrix® Mouse Gene 2.1 ST Array Strip (Affymetrix, MA, USA). RNA was prepared for analysis using Ambion WT Expression Kit (ThermoFisher Scientific, MA, USA) and GeneChip® WT Terminal Labeling Kit (Affymetrix). Briefly, 100 ng of total RNA, containing spiked in Poly-A RNA controls, was used in a reverse transcription reaction to generate first-strand complementary DNA. After second-strand synthesis, double-stranded complementary DNA was used to generate cRNA. cRNA (15 μg) was then used for a second cycle of first-strand cDNA synthesis and the resultant single stranded cDNA (5.5 μg) was fragmented and end-labeled. Size distribution of the fragments was assessed using an Agilent 2100 Bioanalyzer (Agilent Technologies). End-labeled, fragmented cDNA (3.5 μg), was then used in a 150 μL hybridization cocktail containing hybridization controls (GeneAtlas® Hybridization, Wash, and Stain Kit for WT Array Strips, Affymetrix), of which 120 μL were hybridized on array strips for 20 h at 48°C. Standard post hybridization wash and double-stain protocols were used on an Affymetrix GeneAtlas system, followed by scanning of the array strips.

### Microarray data analysis

The arrays were analyzed using Chipster 2 (Kallio et al., 2011) with a custom cdf file in mogene21stmmentrezg.db, as available from Brainarray database (version 17; Sandberg and Larsson, 2007). Following Robust Multi-array Average normalization and biomaRt annotation, differential expression was determined by empirical Bayes two-group test (Smyth, 2004) with Benjamini-Hochberg multiple testing correction and a *P*-value cut-off of 0.05. For further analyses, only genes with fold changes above 1.5 were included. The heatmap was constructed using matrix2png interface (Pavlidis and Noble, 2003). Venn diagram, created using VENNY 2.1 (Oliveros, 2007), was used to identify the genes that were uniquely and commonly expressed in mice infected with different *S. epidermidis* bacterial populations. Gene ontology (GO) terms enrichment was assessed using the Search Tool for the Retrieval of Interacting Genes/Proteins (STRING) (version 10; Franceschini et al., 2013). Only gene-sets passing significance thresholds (*P* < 0.05 with false discovery rate) were selected for further analysis. To reduce redundancy, GO terms found enriched in STRING were reanalyzed by REVIGO (Supek et al., 2011), allowing for small (0.5) similarity, using the species-specific *Mus musculus* database (in order to fine-tune the calculation of semantic distances which rely on information contents of GO terms for this particular species) and SimRel score. The complete list of the genes differentially and uniquely expressed in splenocytes of mice infected with planktonic, biofilm, or biofilm-released cells is available at GEO database repositorium, under the accession number GSE60992.

### Statistical analysis

Statistical analysis was carried out with GraphPad Prism (CA, USA). The normality of the data obtained was evaluated using Kolmogorov–Smirnov test. Accordingly, Kruskal–Wallis and Dunn's multiple comparison tests were applied and data depicted in median of all independent experiments. Differences among groups were considered significant when *P* < 0.05. Statistical differences found between planktonic and biofilm cells phenotype were not indicated as the aim of the study was not to compare the differences between them. Statistical analysis used for microarrays data evaluation is particular and is specified in “microarrays data analysis” subsection.

## Results

### Biofilm-released cells induce a particular gene expression profile on mouse splenocytes

**Figure 1:**
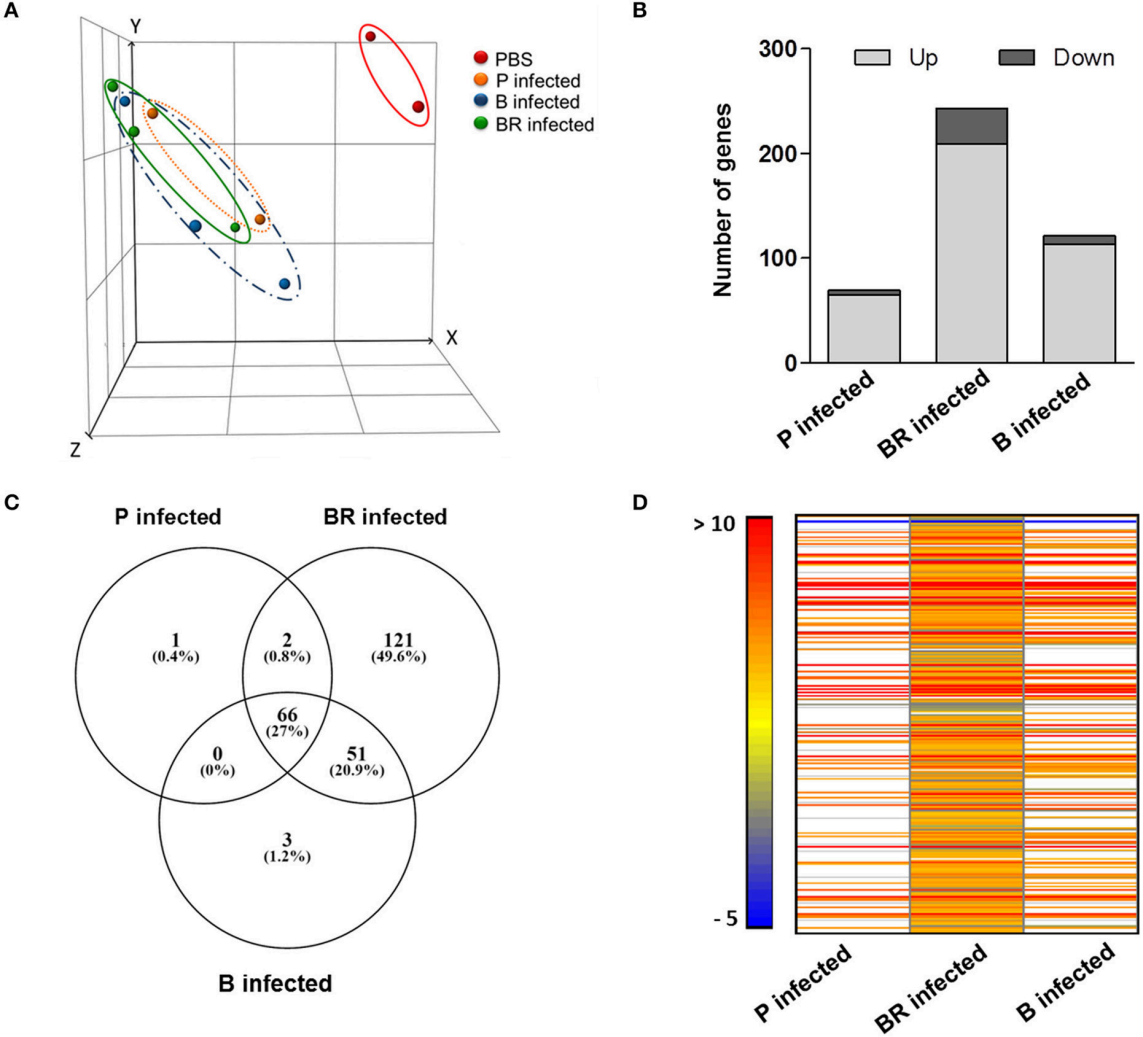
**Analysis of the transcriptome of the spleen of mice infected with different *S. epidermidis* populations**. BALB/c mice were challenged intravenously with 1 × 10^8^ planktonic (P) (*n* = 2), biofilm (B) (*n* = 3), biofilm-released (BR) cells (*n* = 3), or sham-infected treated with PBS alone (PBS) (*n* = 2). Two hours post-infection, spleens were collected and microarray analysis was performed. **(A)** Principal component analysis; **(B)** Number of genes with increased and decreased transcription in each condition (*P* < 0.05, Empirical Bayes two-group test with Benjamini-Hochberg multiple testing correction). **(C)** Venn diagram showing the number of genes that are commonly (overlapping circles) and uniquely expressed (non-overlapping circles) in each condition; **(D)** Heatmap of the differentially expressed genes. White lines indicate non-detected genes or genes with no significant alterations (*P* > 0.05, Empirical Bayes two-group test with Benjamini-Hochberg multiple testing correction).

The transcriptomic profile of mice infected with *S. epidermidis* planktonic, biofilm and biofilm-released cells was compared with that of non-infected mice in order to identify the genes expressed during infection induced by each of the three populations. Principal components analysis revealed that infected mice displayed a markedly different gene expression profile from non-infected controls (Figure 1A). The differences among infected mouse groups, however, were not that evident. The genes with the highest or lowest levels of transcription were similar in the three groups of infected mice (Tables 1, 2). Nevertheless, despite these general similarities important differences were found in the number of genes with increased and decreased transcription (Figure 1B). Within the 243 genes found differentially expressed (*P* < 0.05) in mice infected with biofilm-released cells, 121 were exclusive to this infecting phenotype (Figure 1C), where 96 had increased transcription (above 1.5-fold change) and 25 had decreased transcription (above −1.5-fold change; Figure 1D). Among the genes with increased transcription in splenocytes of mice infected with biofilm-released cells, we found significant enrichment of several GO clusters (Table 3) including positive regulation of leukocyte cell-cell adhesion, tumor necrosis factor-mediated signaling and T cell activation, and negative regulation of mitogen activated protein kinases (MAPK) cascade and interleukin-10 production. Interestingly, GO terms associated with programmed cell death such as regulation of intrinsic apoptotic signaling pathways, development of cell death, and cell killing were also enriched. Finally, we observed that the great majority of the transcripts of the genes with increased expression in mice infected with biofilm-released cells were those encoding proteins mostly localized to the cytoplasm or in cells' organelles (Table 3). No enrichment was found among down-regulated genes, in any of the conditions tested. For further information regarding the GO terms found enriched in mice infected with planktonic or biofilm cells please see Supplementary Material.

**Table 1:** **List of the top most transcribed genes in the spleen of mice infected with *S. epidermidis* planktonic, biofilm or biofilm-released cells**.

**Gene**	**Description**	**Fold change**	***P*-value**
**PLANKTONIC CELLS INFECTED MICE**			
*Irg1*	Immunoresponsive gene 1	39.08 ± 4.02	< 0.001
*Clec4e*	C-type lectin domain family 4, member e	17.46 ± 4.73	0.004
*Cxcl2*	Chemokine (C-X-C motif) ligand 2	16.63 ± 3.64	0.003
*Cxcl10*	Chemokine (C-X-C motif) ligand 10	8.73 ± 2.98	0.012
*Clec5a*	C-type lectin domain family 5, member a	7.80 ± 0.92	0.003
*Slc7a11*	Solute carrier family 7, member 11	7.88 ± 2.47	0.013
*Ccrl2*	Chemokine (C-C motif) receptor-like 2	7.20 ± 0.67	0.006
*Mmrgpra2a*	MAS-related GPR, member A2A	7.15 ± 2.17	0.012
*Gpr84*	G protein-coupled receptor 84	6.31 ± 1.23	0.009
*IIl1rn*	Interleukin 1 receptor antagonist	5.93 ± 0.95	0.008
**BIOFILM-RELEASED CELLS INFECTED MICE**			
*Irg1*	Immunoresponsive gene 1	49.02 ± 5.41	< 0.001
*Cxcl2*	Chemokine (C-X-C motif) ligand 2	25.90 ± 3.85	< 0.001
*Clec4e*	C-type lectin domain family 4, member e	20.24 ± 0.24	< 0.001
*Slc7a11*	Solute carrier family 7, member 11	13.33 ± 1.99	< 0.001
*Mrgpra2a*	MAS-related GPR, member A2A	9.69 ± 0.14	< 0.001
*Ccrl2*	Chemokine (C-C motif) receptor-like 2	8.83 ± 1.03	< 0.001
*Clec5a*	C-type lectin domain family 5, member a	8.52 ± 1.10	< 0.001
*Cxcl10*	Chemokine (C-X-C motif) ligand 10	7.34 ± 0.46	< 0.001
*Fpr1*	Formyl peptide receptor 1	7.22 ± 1.18	< 0.001
*Cd14*	CD14 antigen	7.15 ± 0.47	< 0.001
**BIOFILM CELLS INFECTED MICE**			
*Irg1*	Immunoresponsive gene 1	42.97 ± 4.79	< 0.001
*Clec4e*	C-type lectin domain family 4, member e	18.49 ± 2.67	< 0.001
*Cxcl2*	Chemokine (C-X-C motif) ligand 2	18.22 ± 3.27	< 0.001
*Slc7a11*	Solute carrier family 7, member 11	10.51 ± 2.77	< 0.001
*Cxcl10*	Chemokine (C-X-C motif) ligand 10	10.24 ± 1.37	< 0.001
*Ccrl2*	Chemokine (C-C motif) receptor-like 2	8.54 ± 0.47	< 0.001
*Clec5a*	C-type lectin domain family 5, member a	8.61 ± 1.07	< 0.001
*Mrgpra2a*	MAS-related GPR, member A2A	7.68 ± 0.87	< 0.001
*Fpr1*	Formyl peptide receptor 1	6.59 ± 0.83	< 0.001
*Gpr84*	G protein-coupled receptor 84	6.18 ± 0.37	< 0.001

**Table 2:** **List of the top less transcribed genes in the spleen of mice infected with *S. epidermidis* planktonic, biofilm, or biofilm-released cells**.

**Gene**	**Description**	**Fold change**	***P*-value**
**PLANKTONIC CELLS INFECTED MICE**			
*Abcd2*	ATP-binding cassette, sub-family D (ALD), member 2	−5.20 ± 1.41	0.013
*Kctd12b*	Potassium channel tetramerisation domain containing 12b	−2.14 ± 0.22	0.038
*Mmp12*	Matrix metallopeptidase 12	−2.03 ± 0.15	0.038
**BIOFILM-RELEASED CELLS INFECTED MICE**			
*Abcd2*	ATP-binding cassette, sub-family D (ALD), member 2	−5.31 ± 1.73	0.005
*Ccr2*	Chemokine (C-C motif) receptor 2	−2.43 ± 0.40	0.017
*Sema6a*	Sema domain, transmembrane domain (TM), and cytoplasmic domain, (semaphorin) 6A	−2.38 ± 0.37	0.014
*Kctd12b*	Potassium channel tetramerisation domain containing 12b	−2.35 ± 0.24	0.003
*Pcdha7*	Protocadherin alpha 7	−2.34 ± 0.30	0.005
*Gm3376*	Predicted gene 3376	−2.33 ± 0.23	0.004
*Rgs2*	Regulator of G-protein signaling 2	−2.33 ± 0.39	0.012
*Tlr8*	Toll-like receptor 8	−2.26 ± 0.36	0.028
*Kcnj16*	Potassium inwardly-rectifying channel, subfamily J, member 16	−2.17 ± 0.27	0.009
*Mmp12*	Matrix metallopeptidase 12	−2.18 ± 0.54	0.045
**BIOFILM CELLS INFECTED MICE**			
*Abcd2*	ATP-binding cassette, sub-family D (ALD), member 2	−4.83 ± 1.66	0.012
*Kctd12b*	Potassium channel tetramerisation domain containing 12b	−2.21 ± 0.27	0.013
*Sema6a*	Sema domain, transmembrane domain (TM), and cytoplasmic domain, (semaphorin) 6A	−2.20 ± 0.15	0.012
*Rgs2*	Regulator of G-protein signaling 2	−2.11 ± 0.21	0.012
*Mmp12*	Matrix metallopeptidase 12	−2.07 ± 0.03	0.005
*Prr5l*	Proline rich 5 like	−1.80 ± 0.15	0.040
*Gm13710*	Predicted gene 13710	−1.78 ± 0.06	0.016
*Vstm4*	V-set and transmembrane domain containing 4	−1.76 ± 0.13	0.032

**Table 3:** **GO term enrichment of the genes with increased transcription in the spleen of mice infected with *S. epidermidis* biofilm-released cells**.

**GO ID**	**Cluster representatives**	**N. of genes**	***P*-value**
**BIOLOGICAL PROCESS**			
GO:2001242	Regulation of intrinsic apoptotic signaling pathway	11	< 0.0001
GO:2000116	Regulation of cysteine-type endopeptidase activity	12	< 0.0001
GO:0043409	Negative regulation of MAPK cascade	9	< 0.0001
GO:1903039	Positive regulation of leukocyte cell-cell adhesion	9	< 0.001
GO:0050870	Positive regulation of T cell activation	8	0.001
GO:0044003	Modification by symbiont of host morphology or physiology	4	0.001
GO:0031329	Regulation of cellular catabolic process	15	0.003
GO:0010623	Developmental programmed cell death	4	0.004
GO:0002260	Lymphocyte homeostasis	5	0.005
GO:0031638	Zymogen activation	6	0.006
GO:0006986	Response to unfolded protein	5	0.007
GO:0031341	Regulation of cell killing	5	0.007
GO:0010243	Response to organonitrogen compound	13	0.007
GO:0032693	Negative regulation of interleukin-10 production	3	0.007
GO:1901698	Response to nitrogen compound	14	0.010
GO:0048646	Anatomical structure formation involved in morphogenesis	18	0.010
GO:0019724	B cell mediated immunity	5	0.011
GO:0035966	Response to topologically incorrect protein	5	0.011
GO:0010727	Negative regulation of hydrogen peroxide metabolic process	2	0.011
GO:0010743	Regulation of macrophage derived foam cell differentiation	3	0.014
GO:0043243	Positive regulation of protein complex disassembly	3	0.014
GO:0033043	Regulation of organelle organization	19	0.016
GO:0009628	Response to abiotic stimulus	16	0.017
GO:0009888	Tissue development	25	0.017
GO:0033209	Tumor necrosis factor-mediated signaling pathway	3	0.020
GO:0042940	D-amino acid transport	2	0.023
GO:0016192	Vesicle-mediated transport	16	0.030
GO:0018149	Peptide cross-linking	3	0.030
GO:0045787	Positive regulation of cell cycle	8	0.030
GO:0061028	Establishment of endothelial barrier	3	0.032
GO:0048147	Negative regulation of fibroblast proliferation	3	0.035
GO:0008637	Apoptotic mitochondrial changes	4	0.038
GO:0034976	Response to endoplasmic reticulum stress	5	0.046
GO:0009314	Response to radiation	9	0.046
GO:0051604	Protein maturation	7	0.047
GO:0051195	Negative regulation of cofactor metabolic process	2	0.048
GO:0048661	Positive regulation of smooth muscle cell proliferation	4	0.048
**MOLECULAR PROCESS**			
GO:0050786	RAGE receptor binding	4	< 0.001
GO:0016209	Antioxidant activity	6	0.005
GO:0048020	CCR chemokine receptor binding	4	0.005
GO:0046983	Protein dimerization activity	24	0.016
GO:0038024	Cargo receptor activity	5	0.038
GO:0042803	Protein homodimerization activity	17	0.046
**CELLULAR COMPONENTS**			
GO:0031988	Membrane-bounded vesicle	50	< 0.001
GO:0043226	Organelle	108	0.002
GO:0043227	Membrane-bounded organelle	116	0.016
GO:0044424	Intracellular part	115	0.017
GO:0005737	Cytoplasm	99	0.017
GO:0005912	Adherens junction	12	0.018
GO:0072559	NLRP3 inflammasome complex	2	0.027
GO:0005622	Intracellular	114	0.032
GO:0005576	Extracellular region	46	0.042

Gene set enrichment was primary assessed with STRING (Franceschini et al., 2013) and then the GO terms only found in this condition were analyzed by REVIGO (Supek et al., 2011) to reduce redundancy.

A more comprehensive analysis revealed that within the greatest transcribed genes in mice infected with biofilm-released cells are genes with important functions in both innate and adaptive immune response such as those encoding the early activation marker CD69, and the co-stimulatory molecules CD80, CD86, and CD83, which are expressed on antigen-presenting cells and up-regulated upon exposure to pathogens. Furthermore, mRNA encoding the cytokine CCL17 or TARC (thymus and activation-regulated chemokine), a T cell attractant chemokine produced by dendritic cells, was found significantly up-regulated.

### Biofilm-released cells induce higher stimulation of pro-inflammatory cytokines and chemokines

**Figure 2:**
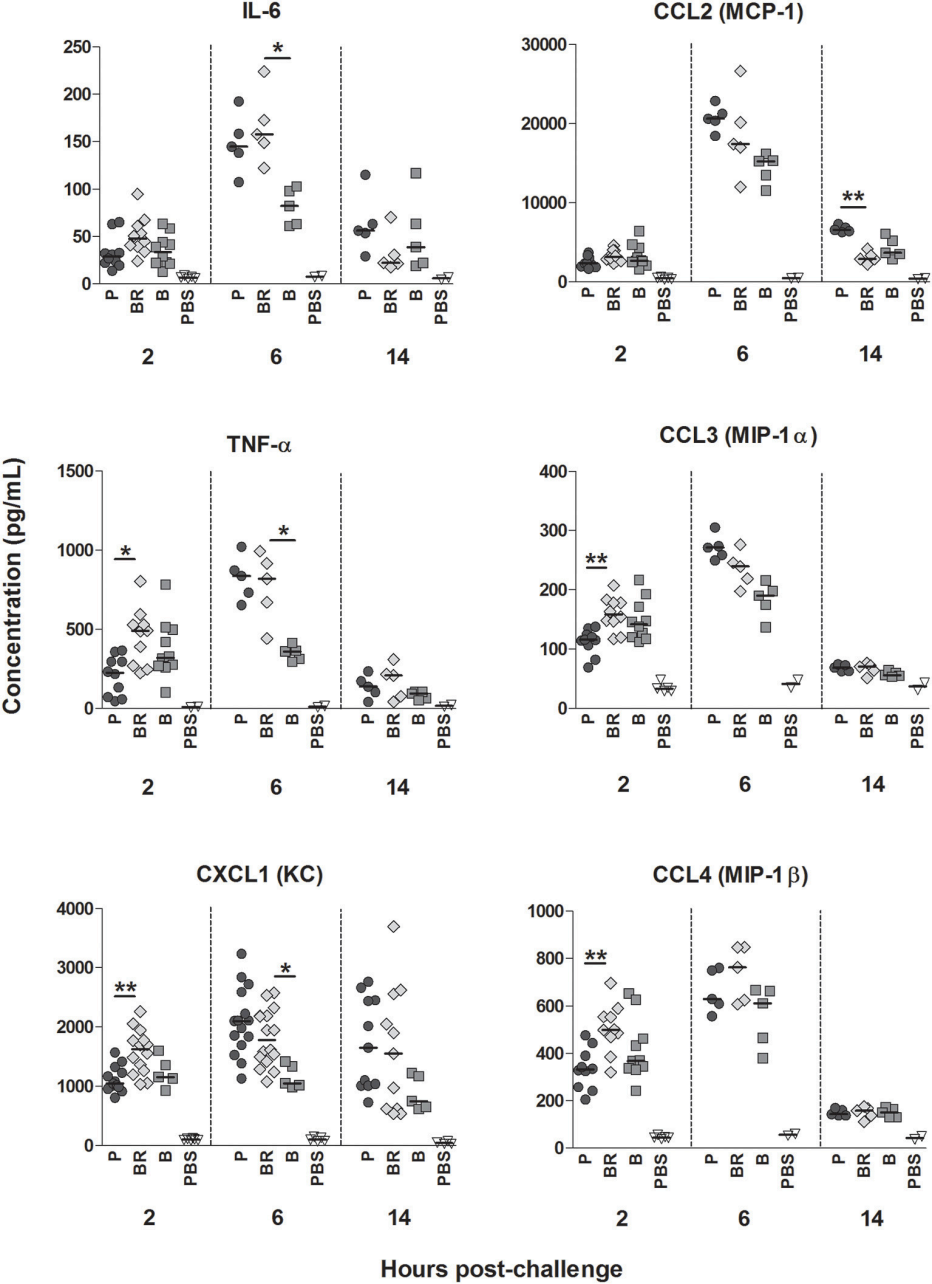
**Pro-inflammatory cytokines and chemokines induced by the different *S. epidermidis* populations**. BALB/c mice were challenged intravenously with 1 × 10^8^ planktonic (P), biofilm (B), biofilm-released (BR) cells, or sham-infected treated with PBS alone (PBS). The serum levels of the indicated cytokines were assessed 2, 6 and 14 h after infection. The obtained results are displayed as the concentration, in ρg/mL, and the horizontal bars represent the median with range of 1 (6 and 14 h time points) to 2 independent (2 h time point) experiments that, per time point, presented the following number of animals: PBS *n* = 2/2/2; P *n* = 10/5/5; BR *n* = 10/5/5; B *n* = 10/5/5. Statistical differences among infected groups were evaluated using Kruskal–Wallis (Overall ANOVA *P* < 0.05) and *post hoc* Dunn's multiple comparison tests. ^*^P < 0.05, ^**^P < 0.01.

As shown in Figure 2, mice infected with biofilm-released cells had significantly higher serum levels of the chemokines CCL3, CCL4, and CXCL1, as well as higher levels of TNF-α than mice infected with planktonic cells, 2 h after the bacterial challenge. At that time point, no differences were found in the levels of any assessed cytokines between biofilm and biofilm-released cells-infected mouse groups. In contrast, 6 h after infection, markedly higher serum levels of CXCL1, TNFα, and IL-6 were detected in mice infected with biofilm-released cells than in the biofilm cell-infected counterparts. By 14 h after infection, lower serum levels of CCL2 were detected in mice infected with biofilm-released cells, when compared with their planktonic infected counterparts. No significant differences were detected in the serum levels of any other assessed cytokine among the different infected groups.

### Biofilm-released cells present an intermediate ability to colonize murine organs

**Figure 3:**
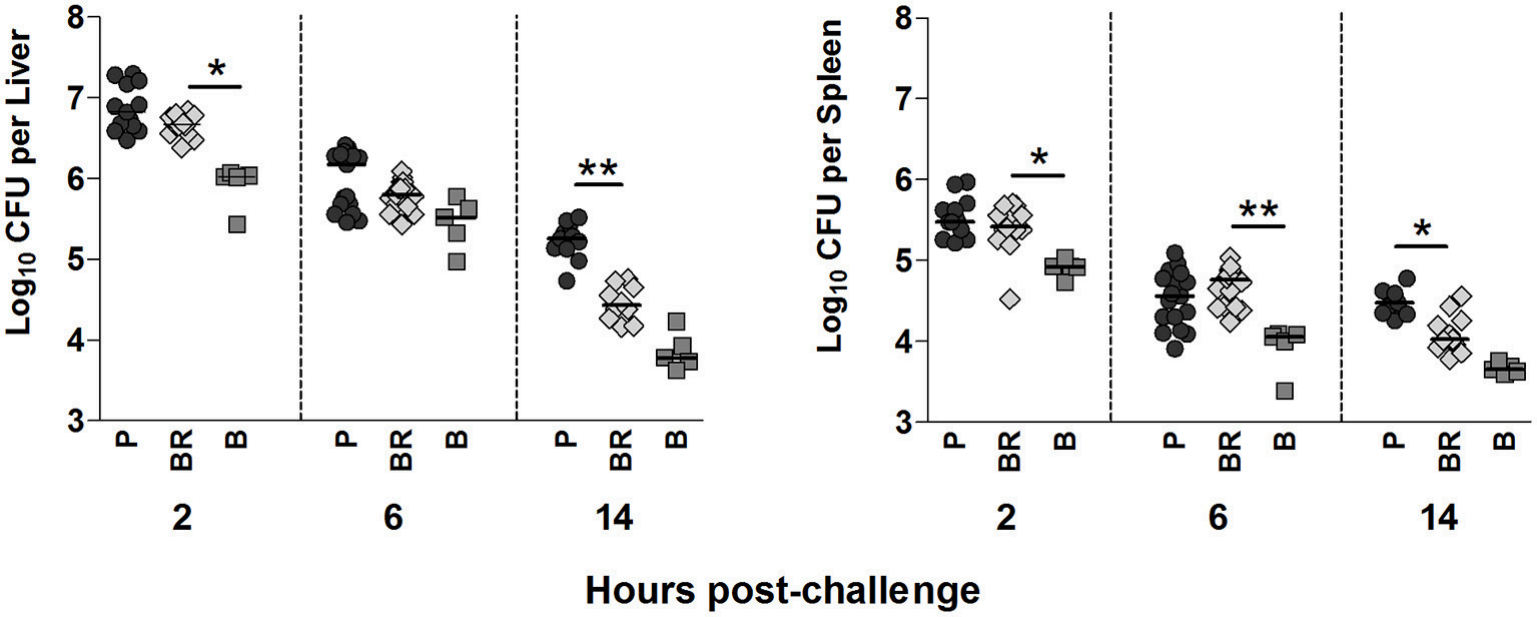
**Liver and spleen bacterial load after infection with the different *S. epidermidis* populations**. BALB/c mice were challenged intravenously with 1 × 10^8^ planktonic (P), biofilm (B), biofilm-released (BR) cells, or sham-infected treated with PBS alone (PBS). Liver and spleen bacterial burden was assessed 2, 6, and 14 h after intravenous infection. Each symbol represents an individual mouse and horizontal bars the median of 1 (biofilms) to 3 (P and BR, 6h time point) independent experiments that, per time point, presented the following number of animals: P *n* = 14/16/11; BR *n* = 14/16/11; B *n* = 5/5/5. Statistical differences among groups were evaluated with Kruskal–Wallis (Overall ANOVA *P* < 0.05) and *post hoc* Dunn's multiple comparison tests. ^*^P < 0.05, ^**^P < 0.01.

Biofilm-released cells had an intermediate ability, between that of planktonic and biofilm cells, to colonize the liver and spleen (Figure 3). Interestingly, while in the first 6 h of infection, biofilm-released cell burden resembled that of planktonic cells, 14 h after infection the differences between planktonic and biofilm-released cells and the similarities between biofilm-released and biofilms cells become noticeable. It is important to note that although the inoculum was adjusted by flow cytometry the quantity of bacteria injected was also confirmed by CFU counting, and the number of CFU was similar among the different populations.

## Discussion

Due to the significant role of biofilms in the emergence of nosocomial infections, previous studies have focused on comparisons between planktonic cultures and established biofilms in order to highlight particular features of biofilm-associated infections (Becker et al., 2001; Resch et al., 2005; Shemesh et al., 2007). The role of biofilm-released cells in the pathogenesis of biofilm infections is, however, poorly understood, with no prior studies addressing this issue in regard to *S. epidermidis* infection. We have recently shown that biofilm-released cells, obtained using the same experimental model used herein, are more tolerant than planktonic cells, or even biofilm cells, to antibiotics commonly used for staphylococcal infections treatment (Franca et al., 2016). Nevertheless, nothing is known about the interplay between these cells and the host immune system. Hence, we have evaluated the interaction between *S. epidermidis* biofilm-released cells and the host immune system, using planktonic and biofilm cells for comparative purposes. We first determined whether biofilm-released cells would induce a different transcriptional profile in splenocytes of mice infected through the hematogenous route. Transcriptomic data showed that mice challenged with biofilm-released cells responded distinctly from the ones infected with the other bacterial populations. Although, a striking difference was observed between control and infected mouse groups, less marked alterations were found within the mouse groups infected with the three *S. epidermidis* populations. Since we compared the response of the host to the same bacterium but in different stages of their lifecycle, fewer differences among infected groups were expected. However, a more exhaustive analysis revealed that the expression level of several genes encoding proteins involved, direct or indirectly, in the development of innate and adaptive immunity were significantly increased in biofilm-released cells-infected mice. An increased transcription of *S100a8* and *S100a9* genes, both encoding damage-associated proteins released mainly by degranulating neutrophils (Simard et al., 2011) and *Ly6g*, which encodes a neutrophil surface marker (Lee et al., 2013) were detected in splenocytes after 2 h of injection of biofilm-released cells. These mice also had the highest expression of *Cxcl2* and *Fpr1* encoding, respectively, neutrophil chemoattractant cytokine CXCL2 (Kobayashi, 2008) and chemotactic receptor formyl peptide receptor 1 that is also present on neutrophil cell membranes (Yang and Hwang, 2016). In accordance with the inflammatory-type response observed in microarrays analysis, these mice also obtained the highest serum levels of neutrophil chemo attractant cytokines CXCL1 and CCL3 (Kobayashi, 2008) 2 h after the challenge of biofilm-released cells. These results indicate that biofilm-released cells may be particularly effective in promoting neutrophil recruitment and activation. Neutrophils are very effective in eliminating extracellular bacteria (Nathan, 2006), and therefore the type and magnitude of response elicited by biofilm-released cells may explain their faster or more effective clearance from the liver and spleen of infected mice, as compared to planktonic cells. Moreover, biofilm-released cells were also more effective at inducing *Irg1* expression, a gene known to be highly expressed in macrophages in response to infections that limits bacterial survival (Cordes et al., 2015). In agreement with the pro-inflammatory response elicited, biofilm-released cell-infected mice showed down-regulated transcription of the anti-inflammatory cytokine interleukin-10. IL-10 is a key cytokine in decreasing inflammatory pathology (Saraiva and O'Garra, 2010), such as that resulting from infection (Duell et al., 2012) by negatively regulating inflammation (Couper et al., 2008). The impact of IL-10 repression in the context of *S. epidermidis* biofilm-released cells bloodstream infections would thus be worth to explore. Nevertheless, mice infected with biofilm cells, were the ones presenting the lowest bacterial burden although not eliciting the highest pro-inflammatory response as could be inferred from gene expression or cytokine levels. A possible explanation for the delayed clearance of biofilm-released cells as compared to biofilm cells may be an enhanced apoptosis of immune cells. This is supported by the significant enrichment of genes associated with this type of cell death observed in mice infected with biofilm-released cells such as the Caspase-4, Caspase-8, and FAS-associated death domain-like apoptosis regulator (Ulett and Adderson, 2006). Furthermore, enrichment of genes related to the assembly of the NLRP3 inflammasome complex, which has been associated in cell apoptosis and pyroptosis (Sagulenko et al., 2013), was also observed. Interestingly, it was recently shown that during early *Mycobacterium avium* biofilm infection, mononuclear cells phagocytic function was attenuated due to hyperstimulation of phagocytes and enhanced cell death by apoptosis induced by biofilm cells (Rose and Bermudez, 2014). Although, we have not specifically addressed this phenomenon in *S. epidermidis* biofilm cells-infected mice, our results suggest that it would be worth investigate in future studies whether biofilm-released cells may employ a similar strategy to circumvent host inflammatory response.

Our results also suggest that biofilm-released cells might be particularly effective in activating antigen-presenting cells, specifically dendritic cells. This hypothesis is based on the significant increase in mRNA encoding the T cell co-stimulatory molecules CD80 and CD86 (Vasilevko et al., 2002; Sansom et al., 2003), the CD83 marker of mature dendritic cells (Lechmann et al., 2008), as well as CCL22 that encodes a chemokine secreted by both macrophages and dendritic cells (Yamashita and Kuroda, 2002) and CCL17 (TARC). Although, CCL17 has been associated with Th2-type responses (Xiao et al., 2003) how biofilm-released cells might affect T cell polarization should be determined in functional assays. In addition, since the spleen comprises cell types other than myeloid cells, including leukocytes and also non-hematopoietic cells, that may be able to produce pro-inflammatory mediators (Fritz and Gommerman, 2011; Bronte and Pittet, 2013), a better characterization of the cells particularly stimulated by biofilm-released cells is needed in order to identify the precise mechanism by which these cells interact with the host immune system.

It is important to emphasize that in this study only one *S. epidermidis* strain was used, and therefore, it was not possible to assess if these observations are transversal to the species or a strain-dependent phenomenon. Moreover, biofilm-released cells distinctive properties, in particular surface antigens, need to be fully characterized as these seem to have important consequences in the outcome of biofilm infections constituting interesting targets. Overall, our results indicate that *S. epidermidis* biofilm-released cells interact distinctly than planktonic or biofilm cells with the host immune system being particularly effective in inducing the production of pro-inflammatory cytokines and in stimulating neutrophils and monocytes. Biofilm-released cells might thus be of particular relevance in inducing deleterious inflammation frequently associated with *S. epidermidis* biofilm infections (Römling and Balsalbre, 2012) highlighting the urgent need to extend the study of *S. epidermidis* biofilm-originated infections by addressing the cells released by biofilms.

Finally, our findings also raise important concerns related to the current strategies proposed for the treatment of staphylococcal biofilm-related infections. The use of matrix-degrading enzymes, such as dispersin B, which is capable of dispersing cells from established biofilms (Kaplan, 2010), is one of the most frequently suggested strategies for the treatment for staphylococcal infections. However, as indicated by the data presented here, the use of matrix-degrading enzymes or other compounds leading to biofilm disassembly need to be carefully considered as biofilm-released cells can heighten the inflammatory response of the host consequently augmenting disease severity.

## Author contributions

NC, GP, and MV designed the experiments. AF, BP, AC carried out the laboratory experiments. AF, GP, MV, and NC analyzed the data, interpreted the results, discussed analyses, interpretation and presentation. AF, AC, GP, NC, and MV wrote the paper. All authors have contributed to, seen and approved the manuscript.

## Funding

This work was supported by European Union funds (FEDER/COMPETE) and by national funds (FCT) under the project with reference FCOMP-01-0124-FEDER-014309 (PTDC/BIA-MIC/113450/2009). The authors thank the FCT Strategic Project of UID/BIO/04469/2013 unit, and the project RECI/BBB-EBI/0179/2012 (FCOMP-01-0124-FEDER-027462). NC is an Investigator FCT. AF is supported by the FCT fellowship SFRH/BPD/99961/2014 and AC by the fellowship SFRH/BPD/91623/2012. The funders had no role in study design, data collection and interpretation, or the decision to submit the work for publication.

### Conflict of interest statement

The authors declare that the research was conducted in the absence of any commercial or financial relationships that could be construed as a potential conflict of interest.
