# Supplementary material for: Staphylococcus epidermidis Biofilm-Released Cells Induce a Prompt and More Marked In vivo Inflammatory-Type Response than Planktonic or Biofilm Cells
Source: Front Microbiol. 2016 Sep 27;7:1530. doi: 10.3389/fmicb.2016.01530 (PMC5037199; doi:10.3389/fmicb.2016.01530)
Supplement: Supplementary file 1 [file Table1.docx]

Supplementary Material

# ***Staphylococcus epidermidis* biofilm-released cells induce a prompt and more marked *in vivo* inflammatory-type response than planktonic or biofilm cells**

Angela França, Begoña Perez-Cabezas, Alexandra Correia, Gerald B. Pier, Nuno Cerca*, Manuel Vilanova

*** Correspondence:** Nuno Cerca: [nunocerca@ceb.uminho.pt](mailto:nunocerca@ceb.uminho.pt)

# Supplementary Figures and Tables

## Supplementary Tables

**Supplementary Table 1.** Gene ontology term enrichment of the genes with increased transcription in the spleen of mice infected with *S. epidermidis* planktonic cells. Gene set enrichment was primary assessed with STRING and then the GO terms only found in this conditions were analysed by REVIGO to reduce redundancy.

| **GO ID** | **Cluster representatives** | **Nº genes** | ***P* value** |
| --- | --- | --- | --- |
| **Biological process** | | | |
| GO:0022408 | Negative regulation of cell-cell adhesion | 4 | 0.021 |
| GO:0002335 | Mature B cell differentiation | 2 | 0.030 |
| GO:0034123 | Positive regulation of toll-like receptor signaling pathway | 2 | 0.030 |
| GO:0032703 | Negative regulation of interleukin-2 production | 2 | 0.033 |
| GO:0048878 | Chemical homeostasis | 8 | 0.037 |
| GO:1901741 | Positive regulation of myoblast fusion | 2 | 0.040 |
| GO:0042107 | Cytokine metabolic process | 2 | 0.044 |
| GO:0003085 | Negative regulation of systemic arterial blood pressure | 2 | 0.048 |

**Supplementary Table 2.** Gene ontology term enrichment of the genes with increased transcription in the spleen of mice infected with *S. epidermidis* biofilm cells. Gene set enrichment was primary assessed with STRING and then the GO terms only found in these conditions were analysed by REVIGO to reduce redundancy.

| **GO ID** | **Cluster representatives** | | **Nº genes** | | | ***P* value** | |
| --- | --- | --- | --- | --- | --- | --- | --- |
| **Biological process** | | | | | | | |
| GO:0045637 | Regulation of myeloid cell differentiation | | | | 7 | 0.001 | |
| GO:0032844 | Regulation of homeostatic process | | | | 8 | 0.010 | |
| GO:0000302 | Response to reactive oxygen species | | | | 5 | 0.010 | |
| GO:0046890 | Regulation of lipid biosynthetic process | | | | 5 | 0.012 | |
| GO:0048525 | Negative regulation of viral process | | | | 4 | 0.014 | |
| GO:0051952 | Regulation of amine transport | | | | 4 | 0.015 | |
| GO:0001952 | Regulation of cell-matrix adhesion | | | | 4 | 0.017 | |
| GO:0045786 | Negative regulation of cell cycle | | | | 7 | 0.018 | |
| GO:0048662 | Negative regulation of smooth muscle cell proliferation | | | | 3 | 0.020 | |
| GO:0032757 | Positive regulation of interleukin-8 production | | | | 3 | 0.020 | |
| GO:0032768 | Regulation of monooxygenase activity | | | | 3 | 0.020 | |
| GO:0050829 | Defense response to Gram-negative bacterium | | | | 3 | 0.020 | |
| GO:2000351 | Regulation of endothelial cell apoptotic process | | | | 3 | 0.021 | |
| GO:0042307 | Positive regulation of protein import into nucleus | | | | 4 | 0.025 | |
| GO:0034383 | Low-density lipoprotein particle clearance | | | | 2 | 0.025 | |
| GO:0019233 | Sensory perception of pain | | | | 4 | 0.026 | |
| GO:2001237 | Negative regulation of extrinsic apoptotic signaling pathway | | | | 4 | 0.026 | |
| GO:0071801 | Regulation of podosome assembly | | | | 2 | 0.030 | |
| GO:1901998 | Toxin transport | | | | 3 | 0.031 | |
| GO:0032495 | Response to muramyl dipeptide | | | | 2 | 0.034 | |
| GO:0006684 | Sphingomyelin metabolic process | | | | 2 | 0.034 | |
| GO:0030656 | Regulation of vitamin metabolic process | | | | 2 | 0.034 | |
| GO:0006979 | Response to oxidative stress | | | | 6 | 0.048 | |
| **Molecular process** | | | | | | | |
| GO:0001614 | | Purinergic nucleotide receptor activity | | 3 | | | 0.048 |
| **Cellular components** | | | | | | | |
| GO:0016021 | | Integral component of membrane | | 44 | | | 0.020 |
| GO:0045121 | | Membrane raft | | 7 | | | 0.048 |
